# How do medical professionals justify their involvement with live tissue training?

**DOI:** 10.1186/s12910-025-01304-3

**Published:** 2025-10-08

**Authors:** C. S. Swain, G. Helgesson

**Affiliations:** 1 Department of Learning, Informatics, Management & Ethics (LIME), Karolinska Institutet, Stockholm, Sweden; 2 Academic Department of Military Surgery & Trauma, Royal Centre for Defence Medicine, Birmingham, UK

**Keywords:** Simulation, Trauma surgery, Animal ethics, Consequentialism

## Abstract

**Background:**

“Live tissue training” (LTT) is simulation that uses a live anaesthetised animal in place of a human patient. It is a training practice which is significantly contested, but continues to occur despite availability of alternative simulator models. The aim of this study was to explore if, and how, medical professionals who participate in LTT justify their own professional involvement.

**Methods:**

Fifteen semi-structured interviews of physicians who had knowledge and prior experience of LTT were performed as part of a wider research project and initially analysed using the Framework Method. Data categorised as ‘ethical views’ underwent a secondary thematic analysis to answer this research aim. Data were grouped by similar meaning to produce themes in the form of beliefs or views expressed by the participants.

**Results:**

Although no participant used language to explicitly indicate moral theorising, there is a set of identifiable coherent beliefs/views among the cohort. A belief that training must be conducted in order to save human patients’ lives (1); that human life is of higher value than animal life (2); and that there is no sufficiently good alternative to LTT (3). It is felt that LTT is reasonable as the numbers of animals used are minimised and opportunities for learning or other uses maximised (4); animals are well cared for and are not suffering (5) and reasonable in comparison to other animal uses (6).

**Conclusion:**

There is a predominant consequentialist thinking regarding the use of live animals, with evidence that the 3Rs principles are being considered, if not explicitly, as a restriction on the use of animals for LTT and also partly to justify or defend medical professionals’ involvement. We suggest that professional identity is likely to have a role in forming these justificatory arguments, but personal views about the moral standing of animals and notions of speciesism could also influence decisions about being involved in LTT.

**Supplementary Information:**

The online version contains supplementary material available at 10.1186/s12910-025-01304-3.

## Background

Good education is key to high quality health care. Simulation is considered a complementary educational technique, used to mitigate the potential harm caused to patients by medical professionals in training [1, 2]. Within healthcare, there is an ethical imperative for patients to be provided with high standards of care, while providing constructive learning opportunities for trainees [1, 2]. 

“Live tissue training” (LTT) is simulation that uses a live anaesthetised animal in place of a human patient. Animals have been used worldwide throughout history as a means to increase understanding of anatomy, physiology and pathology, as well as to improve techniques to treat human disease [3–6], Medical specialties such as surgery [7, 8], anaesthesia [9, 10] and emergency medicine [11, 12] have used live animals to practice procedural skills, although their use has been reduced and completely replaced in some cases, such as the Advanced Trauma Life Support program [13]. Continued use of live animals in surgical education has been contested [5, 6], and increasingly so in recent years.

There are different possible ethical positions regarding such use of animals. According to one deontological approach defending animal rights, with proponents such as Tom Regan, animals have moral rights and must not be treated as mere resources by humans [14]. This is a stance shared by PETA and other animal activist organisations, which, if accepted, means that LTT is unacceptable and should never be undertaken. There are opposing deontological views, such as that disseminated by Carl Cohen, tying being a moral subject to being a moral agent [15]. A belief that animals are not moral agents and therefore have no rights at all would allow LTT to be conducted.

A consequentialist approach values actions in terms of consequences, summarised by the colloquial phrase ‘the end justifies the means’ and with the utilitarian motto ‘maximize wellbeing’. The most prominent proponent of a utilitarian animal protection view is Peter Singer [16], who argues, for instance, that the modern meat industry causes an unacceptable amount of animal suffering to be justifiable and therefore advocates vegetarianism. Therefore, one might expect this approach to advocate for a move towards educational substitutes for LTT. An alternative consequentialist position may be that use of animals – be it hunting for sport, breeding livestock, testing vaccines etc. – in fact has overall positive consequences, but requires that animals are treated well and managed within existing legislation [17]. Both of these consequentialist approaches may inform educators’ decisions about whether or not to use live animals within their training programs.

Much international legislation governing the use of animals is based on an ethical framework for improving laboratory animal welfare, known as the 3Rs. Published by Russell and Burch in ‘The Principles of Humane Experimental Technique’ [18] in 1959, the 3Rs have since been adopted worldwide. Described in an order that reflected the sequence in which they should be addressed, they have remained largely unchanged over time [19]. The 3Rs principles are as follows:

- *Replacement*: substituting sentient animals with insentient material whenever equally good results can be achieved that way.
- *Reduction*: reducing the number of animals used to obtain information to a necessary minimum.
- *Refinement*: decreasing the severity of inhumane procedures (limiting pain and distress) applied to those animals that still must be used.

Educational programs that use live animals must obtain appropriate permissions through a university or governmental ethics board (or equivalent), which generally requires evidence that the training is necessary and that the 3Rs principles are being complied with.

While it was stated already twenty years ago that alternative simulators could supplant live animals [1, 6], LTT continues in surgical education, as many believe that there is not yet a suitable replacement [20]. Alternatives include, but are not limited to: animal or human cadavers, computer technologies, anatomical mannequins, and supervised clinical practice on human patients. A summary of literature examining the pros and cons relating to LTT identified that there are potential, but unverified, advantages relating to the realistic nature of training and opportunities to rehearse situations involving rare and critical traumatic injuries [21]. The review concluded that overall justification regarding the use of live animals in trauma surgical training is unclear. The aim of this study was to explore if, and how, medical professionals who participate in LTT justify their own professional involvement.

## Methodology

Medical professionals who had participated in live tissue training in the role of learner or educator were recruited to contribute to an interview study, as part of a wider doctoral research project conducted by CS about the educational use of LTT [22]. The original intention for this study was exploring the perspectives of learners and educators involved with this type of training to answer the research question “How can LTT be optimised as an educational learning event?” [23] During analysis, it was noted that several perspectives relating to how LTT is, or should be, used educationally were informed by, or intimately connected with, moral or ethical opinions. This led to the idea for a sub-study exploring a different research question: “How do medical professionals justify their involvement with LTT?”

Participants had been recruited from LTT courses observed by CS and via an invitation disseminated by the Royal College of Surgeons of England. The interview protocol is included as supplementary information. Although ethical implications of training were one of the question themes, the majority of participants offered and discussed risks, drawbacks and ethical considerations of live animal training, without direct questioning. For participants who did not spontaneously contribute their views, they were explicitly asked whether they considered there to be any ethical implications associated with LTT. Their expressed ideas were explored in accordance with a semi-structured interview approach, using open-ended questioning.

Fifteen interviews took place between April 2023 and February 2024. The cohort consisted of 13 surgeons, one anaesthetist and one emergency physician, of which four were in active military employment. The majority were consultants (*n* = 13) and male (*n* = 11), working in UK (*n* = 7), Sweden (*n* = 5), or USA (*n* = 2); one participant worked for an international organisation. For nine participants, their main experience of LTT was in the role of learner (reported with numbers 1–9), with six experiencing LTT as educators (reported as letters A-F). Six participants had been involved with LTT (in either role) on three or more occasions.

Transcribed data were initially analysed using the Framework Method [24] during the original study [23]. Within the framework matrix, data were categorised as ‘ethical views’ and it was noted that this was the only category to which every participant contributed data. These coded data were extracted for this sub-study and the interviews were reviewed a second time by CS considering the new research question. Using the process of reflexive thematic analysis [25], CS re-familiarised herself with the data, contextualising it within the wider interview as necessary. Segments of data that demonstrated language indicative of ethics, morals, rationalisation or justification were identified in response to the research question and coded semantically. Data were grouped by similar meaning to produce initial themes, in the form of beliefs or views expressed by the participants. The coded data and formulated themes were discussed with GH, iteratively refined and ordered to form a sequential line of argumentation. Quotes most representative of each thematic viewpoint within the sequence were chosen to aid understanding, with words in italics used by the authors to highlight particular phrases relating to values, morals and ethics.

CS is a military surgeon with an academic interest in medical education and simulation and GH is a philosopher of medical ethics. CS has a knowledge of surgical culture and experience, and through doctoral research, has observed LTT in a variety of settings. The shared cultural understanding assisted with interpretation of context and language both during the interviews and the analysis process. CS holds a neutral stance regarding the use of LTT, considered herself to be neither for nor against the practice in principle. GH was unaware of the practice of LTT prior to his supervisory involvement with this doctoral research project, and has no firsthand experience to inform considerations of whether LTT is ethically justifiable.

## Findings

Although no participant used language to explicitly indicate their moral theorising, or brought up the full sequence of views, there is a set of coherent beliefs/views identifiable within the data. Also, although not explicitly expressed as such, many of the views expressed align with the principles of the 3Rs, demonstrating that participants hold moral views consistent with these ‘moral pillars’ of animal research ethics.

### The belief that training in surgical trauma practices must be conducted in order to save human patients’ lives and improve their outcomes

A prominent line of reasoning expressed by the participants points to the need for this specific professional education in order to be able to provide adequate care to human patients.

- “We need some way to practice, we need some way to ensure that our service personnel, when they’re put in harm’s way for their country, can have the best treatment plans.” (Learner 9).
- “If there are things that we’re doing on humans, then we should try and be *as good as we can be* before we get there.” (Learner 4).
- “This is a difficult situation you put people in, when penetrating trauma happens to a person. And you have no chance to train that beforehand. It is *not fair* to either the patient or the doctor, I will say. [LTT] is a way we can train it.” (Educator E).

### The belief that human life is of higher value than animal life

Secondly, responses clearly suggest that there is an understanding among respondents that human life has priority over animal life.

- “The reason why we use these live animals is so another human could live, or be potentially allowed to live through sets of skills that you learn in that context. […] If killing another animal, not human, serves to benefit humanity. My opinion, *it’s worth it*.” (Educator C).
- “If [LTT] gives you the skills to be able to deal with, you know, my family, when they come into hospital, then yeah, I don’t have a problem with that at all.” (Learner 8).
- “If I’m going to be doing these sorts of things on humans, I would prefer to practice on a pig, rightly or wrongly, beforehand.” (Learner 4).

Among our participants, however, not all animals are considered equally. One participant (Educator C) commented at length about the sentience of different animals (dolphins, chimpanzees, pigs – “extreme – are goldfish sentient animals? Are grasshoppers sentient animals, are worms?”) seemingly inferring that the presence of sentience should determine whether or not they should be used for human purposes. Perhaps unusually among the surgical community, due to the prevalence of cadaveric dissection during training, one participant (Learner 1) expressed a view that “it’s easier to work on a living animal than a dead person […] I think it’s a little bit disrespectful. Even though I know that the people have given their will and so on. But I think, it makes me very uncomfortable.”

### The belief that there is no sufficiently good alternative to live tissue training, neither in terms of appropriate simulator (yet) nor sufficient exposure in clinical practice

Apart from thinking that specific surgical training in the management of traumatic injuries was necessary for future surgical success, and that animals have less value than humans, an additional view was that there is currently no alternative option that could replace the educational experience provided by LTT. Although the first principle among the 3Rs – replacement – was defended, the expressed view appears to be that LTT is justifiable until an appropriate alternative model can be used to achieve the same learning objectives.

- “I agree to the rules that we have, that [LTT] should be used only when we have no other models that are better.” (Educator E).
- “If it’s possible to get the same degree of learning without killing an animal, then yeah, we should, but prove that to me.” (Educator C).
- “I’m afraid until we come up with a better way of being able to simulate the beat-to-beat changes in physiology that an animal model gives…” (Educator B).
- “I think until someone comes up with an artificial model that bleeds like people, that has human-type reactions, then [LTT] is the closest you’ve got to it.” (Learner 9).

### LTT is reasonable as the number of animals used are minimised and opportunities for learning or other uses maximised

In addition to supporting the replacement of animals when possible, the idea of reducing the number of animals used in LTT is expressed by the participants, for instance, in relation to using animals only for certain learning objectives and maximising the amount of learning that can be obtained from a single animal.

- “I think you’ve got to be careful about the ethics of just, you know, going through loads of animals […] you’re going to need to try to use one animal for as much as you can.” (Educator F).
- “I was quite vocal about ATLS [Advanced Trauma Life Support], when they were using the full animal to do a chest drain, I mean it was ridiculous […] I don’t need the animal alive to make a chest drain.” (Educator A).
- “I feel bad when we have an animal lab and don’t use it in the correct way. We could have done this without this animal. […] The more advanced and the more bleeding is involved, it’s *easier to justify* it, I would say.” (Educator E).

### LTT is reasonable because the animals are well cared for and are not suffering

Considerations of the kind covered by the third 3Rs principle – refinement – are also present in the reasoning regarding LTT. The participants demonstrate a belief that the animals had a good quality of life prior to being used for LTT and that being anaesthetised and then euthanised equates to a reasonable and respectful death in comparison to animals in other circumstances.

- “It’s bad for the pig, I’m sure, but they’re all euthanised. None of them wake up to suffer pain. They’re all looked after very well beforehand.” (Learner 4).
- “It was clear that the animals were appropriately anaesthetised, so they were not suffering and in pain, which I think is, humane obviously.” (Educator C).
- “The animals are very, very well looked after in the moment. […] I was really pleasantly surprised at the respect they showed to the animals.” (Learner 8).
- “These animals, they live a short life, but they have a good environment, they’re treated better than most other animals, actually.” (Educator D).

### LTT is reasonable in comparison to other animal uses, such as for food production

One specific line of reasoning, apart from ‘the training is needed’ and ‘animals have lower standing than humans’, is associated with alternative animal uses. Despite recognising that the 3Rs need to be considered, participants were still open to defend LTT in comparison with other uses of animals, not least for meat production. Many participants further compared their involvement with LTT against their own dietary choices.

- “I have no problem when I calculate the numbers of animals used for [LTT], compared to the number of animals we use in the food industry.” (Educator E).
- “Animals do get killed to be eaten. So… being killed to be used for learning, I don’t necessarily think as a *bad thing*.” (Learner 7).
- “I think it would be entirely hypocritical for me as a meat eater… to insist that there was a *moral platform* for me to stand on to say that we shouldn’t be using animals for improving patient outcomes.” (Educator B).
- “I’m born vegetarian (laughs). My teenage self would not forgive me […] I don’t think of it as ethically different from eating animals […] I think it’s interesting that everyone feels that it’s a big difference.” (Learner 2).
- “In the sphere of how we use animals and our relationship with animals, it’s on the *noble side* of things, as opposed to, bacon sandwich and brown sauce kind of things.” (Learner 8).

However, the strength of this better-in-comparison argument was questioned by one participant who stated: “I feel like there’s no moral or ethical grounds to conduct this kind of training or even eating meats, because it’s not necessary and obviously, animals are suffering.” (Learner 5) Interestingly, this participant, who was not a surgeon, reported that while they found their involvement in LTT to be a valuable learning experience, in their opinion, clinical exposure was of greater educational value.

## Discussion

The findings from interviews with medical professionals who had participated in live tissue training, either as learners or educators, show that these participants did perceive there to be moral issues involved in this use of animals for surgical training. Phrases associated with justice and morality, along with value-laden terminology, are used by participants throughout, i.e. good and bad, right and wrong. Comments such as “operating on any live animal is sad”, “it’s bad for the pig” and referring to the animal’s death as a “sacrifice” demonstrate that those involved recognise the moral quandry. However, for a number of respondents, LTT is also considered “easier to justify” as it is for “a noble cause”. Most, but not all, respondents were willing to support the use of LTT to improve surgical skills, as a means to save human lives and improve the health and wellbeing for those in need of surgical treatment.

Although no individual participant went through all stages, in this manner, during their interview, the collective argument by this cohort of medical professionals justifying their involvement with LTT proceeds like this: The kind of surgical trauma training provided by LTT is much needed. Use of animals for this purpose is justified because human beings have greater moral significance than (other) animals, and there is yet no alternative simulation model that can replace LTT. This being said, opportunities to replace LTT with other training modalities need to be considered for each specific use. The potential to reduce the number of animals used and to refine the procedures when using those animals that cannot be replaced must also be considered. Finally, in comparison with other uses of animals, such as for meat, LTT is easier to defend, both in terms of numbers used and in quality of life for the animals used. Hence, without explicitly stating the principles of the 3Rs, it was clear that several of the participants’ views aligned with them.

Regarding the final stage of the argument, that of comparing LTT with other uses of animals, it was noted by one participant that argument by analogy makes a case if there is one practice you gladly accept (like meat eating) and another one is questioned (like LTT) while they in fact are similar in some important respect, then both should be considered comparably. This argument nevertheless does not prove the point, since both practices may be wrong even if one is worse than the other.

There is a limited body of evidence which has explored the educational use of live animals in comparison to another type of simulator, but conclusions relating to primacy of any one modality, especially in relation to trauma training, are not robustly supported [26–28]. A need for training in absence of clinical exposure [7, 29], a lack of suitable alternatives to live animals [7, 29, 30] and a justification for LTT in relation to the food industry [30] have been reported by other studies. It has been argued that LTT is ethically unjustified [31]. However, medical professionals continue to be involved with it and argue for its justification.

### What informs the views of medical professionals?

A question to raise is what informs this argumentation of medical professionals? One potential understanding is that the views expressed by the interview participants reflect broad values in society. Another option is that expressed views are personal without such connections to broadly held views. While not dismissing these explanations, we want to suggest that the beliefs regarding the need for trauma training and the value of human life is likely associated with inherent tenets of medical professionalism.

Medical professionalism is defined as a set of core beliefs and values that guides the behaviour of physicians caring for patients [32, 33]. The profession holds an ethical outlook that is traditionally deontological and patient-centred [34], with a long history of oaths, codes and moral motivations, most notably starting with the Hippocratic Oath in approximately 400 BC [35]. Although it is debated exactly what the profession’s beliefs and values are, some suggestions include respect, altruism, integrity, honesty, compassion and empathy [33]. While resting on deontological pillars, medical practice is also permeated by consequentialist considerations of what is best for the patient.

Teaching professionalism is challenging [36]. Medical professionals typically learn and embrace professional values through a process of socialisation [32], developing their professional identities through interactions with various sociocultural materials [37, 38]. Therefore, medical education encompasses both learning to practice medicine and becoming a doctor [37, 38]. This formation of professional identity is an adaptive, developmental process occurring at the individual and collective level. Evolving throughout medical training in response to new roles and responsibilities [39], one’s professional identity is shaped by challenges and struggles which trigger reflection on their own perspectives and values [39, 40]. Shared culture, including professional standards, responsibilities and moral norms, bind groups together. When individuals become doctors, starting during education, they adopt a shared attitude that saving human life is critically important; this rests on the medical ethical principle of beneficence – the obligation of a physician to act for the benefit of the patient.

Similarly, surgical training is as much about becoming a surgeon as it is about learning to do surgery. The principle of non-maleficence expresses the obligation of a physician to not harm the patient, therefore there is an ethical tension in surgical training, as trainees must practice their skills in a system caring for human patients. The potential for harm, especially in the case of trauma patients, is significant due to the critical nature of the injuries and relatively rare opportunity for practice. Simulation is one technique used to mitigate this risk of harm [34].

The focus on beneficence and non-maleficence became apparent in relation to LTT with the use of phrases such as “weighing a cost benefit… the ethical cost towards the benefit of future patients” (Learner 2), “the risk versus the benefit.” (Learner 8) and “this is for the greater good” (Educator D). The idea of the primacy of human life over animal life, manifest among the participants, may relate to wider ethical positions, but the belief is likely reinforced by their overarching professional identity and sense of duty to help their patients.

Professional identity formation is complex, and individuals may be influenced or conflicted by pre-existing beliefs, cultural or religious associations and wider societal norms [38, 40]. This may, for example, explain one participant’s view that training using a live animal was easier in comparison to using a cadaver, which they felt to be disrespectful. Considering that cadaveric training is a longstanding and highly valued aspect of surgical training [41], this view could be considered odd amongst this professional group. However, the status of a dead body is considered markedly different to that of a dead animal, upholding the idea of human dignity [42].

### A range of beliefs

Acceptance of LTT within the medical and academic communities is seemingly higher than that of the general public [7, 43]. Senior individuals within these professions are more likely to agree with, and justify, live animal use [43], which could perhaps indicate socialisation of professional culture. Interestingly, LTT is considered acceptable even when individuals within the medical profession have no personal experience of the training [7, 43]. 

However, even within this small sample of medical professionals, there was a spectrum of beliefs in relation to specific use of animals as educational instruments. One participant commented that “it was unfair that we had the bad pig” (Learner 1) in relation to the fact that the animal had died (due to poor health) at the start of the training day. This unfairness was interpreted as a loss of beneficial educational opportunity rather than an issue of moral concern for the wellbeing of the pig. Another participant stated clearly that “animals are suffering” (Learner 5) surmising that there was no moral or ethical grounds to conduct LTT or eat meat, and therefore they did not intend to participate in LTT again, despite conceding that their learning experience was a beneficial one.

A wider societal view about the moral status of people and animals was reflected in some of the participant’s statements, for example one participant (Learner 3) commented “if there was a dog or something, I would 100% not participate”. Shelly Kagan holds a hierarchical view that people and animals have different moral status, with some animals ranked higher or lower based on perceived intelligence and psychological capacities [44]. Notably in Kagan’s suggested hierarchy, both dogs and pigs are grouped together as ‘highly intelligent animals’. However in Western cultures, dogs are often pets and treated as a member of the family, whereas pigs are predominantly considered as a source of food and looked upon as less worthy of consideration regarding their wellbeing; this is known as pet speciesism [45, 46]. 

The concept of the human-animal bond is evidentially supported in veterinary medicine [3]. Pet owners may take considerable measures, at great financial cost, to save or heal long-loved pets, while commercial considerations tend to decide the outcome in decisions regarding, e.g. diseased pigs, sheep, or cows. This provides reason to think that emotional bonds rather than more thought-through philosophical positions on intrinsic value and moral standing guide decisions to treat pets as more important [42]. Individuals are likely to be more opposed to live animal use if they have a bond with the species and are concerned about pain and suffering [43, 46]. 

While professional culture may influence the justification argument relating to LTT, there are wider factors relating to the individual, the choice of animal, their personal involvement with that species and how the animal is used [46], which may further inform an individual’s decision or not to be involved with this type of training.

### Limitations

Recognising that there are variable moral and cultural views on the use of animals for human purposes and their treatment, we are not trying to suggest that the views presented here are universally generalisable. This small study population of fifteen physicians has a male gender bias and are located within high-income Western countries; it is possible that physicians with other demographics may hold different perspectives. Religious views may inform opinions, however these were neither offered by our participants, nor explored. Additionally, the study recruited participants with experiential knowledge of LTT, the opinions of those who have not, or would not, participate in LTT may provide opposing or alternative views.

## Conclusion

This analysis of interview data collated from medical professionals involved in LTT indicates a predominant consequentialist thinking regarding the use of live animals, combined with an understanding that the wellbeing of humans counts for more than the wellbeing of animals. There is evidence that the 3Rs principles are being considered, if not explicitly, as a restriction on the use of animals for LTT and also partly to justify or defend their involvement. We suggest that professional identity is likely to have a role in forming these justificatory arguments, but personal views about the moral standing of animals and notions of speciesism could also influence decisions about being involved in LTT.

Justifiability is likely to exist on a spectrum, but the requirement to address the 3Rs principles in surgical education is fixed. Replacement is the ultimate goal, but the belief that there is no suitable alternative to live animals is an issue. Arguably, educators and other members of the community should continually be considering whether live animal use is appropriate or required and actively be exploring non-animal options, to replace all or part of the training. Clear justification regarding LTT with supporting evidence of beneficial educational outcomes is important to strive towards, rather than trusting in the traditional established practice of a medical professional culture.

## Supplementary Information

Supplementary Material 1.

## Data Availability

The datasets used and/or analysed during the current study are available from the corresponding author on reasonable request.
